# Corrigendum to “Probing for Sparse and Fast Variable Selection with Model-Based Boosting”

**DOI:** 10.1155/2018/2430438

**Published:** 2018-07-05

**Authors:** Janek Thomas, Tobias Hepp, Andreas Mayr, Bernd Bischl

**Affiliations:** ^1^Department of Statistics, LMU München, München, Germany; ^2^Department of Medical Informatics, Biometry and Epidemiology, FAU Erlangen-Nürnberg, Erlangen, Germany; ^3^Department of Medical Biometry, Informatics and Epidemiology, University Hospital Bonn, Bonn, Germany

In the article titled “Probing for Sparse and Fast Variable Selection with Model-Based Boosting” [1], an Acknowledgment should be added as follows:
